# Metachronous rectal metastasis from primary transverse colon cancer: a case report

**DOI:** 10.1186/s40792-018-0498-0

**Published:** 2018-08-09

**Authors:** Shigehiro Kojima, Tsuguo Sakamoto, Yuko Nagai, Masayuki Honda, Fumihiro Ogawa

**Affiliations:** 1 Department of Surgery, Sainokuni Higashiomiya Medical Center, 1522 Toro-cho, Kita-ku, Saitama, 331-0804 Japan; 2 Department of Pathology, Sainokuni Higashiomiya Medical Center, Saitama, Japan

**Keywords:** Colorectal carcinoma, Gastrointestinal metastasis, Metastatic colorectal cancer

## Abstract

**Background:**

Colorectal metastases from primary colorectal cancers are very rare, and little is known about their epidemiological aspects or the best diagnostic and therapeutic strategies. Herein, we report a case of a 65-year-old woman with suspected metachronous metastasis to the rectum from primary transverse colon cancer.

**Case presentation:**

The patient underwent a laparoscopic extended right hemicolectomy for primary transverse colon cancer. Histopathological examination showed moderately differentiated adenocarcinoma, and the tumor was diagnosed as stage IIA (T3, N0, M0). Fifteen months after her colectomy, a computed tomography scan demonstrated a rectal tumor and a right ovarian tumor. Colonoscopy revealed a superficial elevated lesion in the middle rectum, and histological analysis showed moderately differentiated adenocarcinoma. Laparoscopic low anterior resection preserving the left colic artery and bilateral adnexectomy were performed. Histological examination of the rectal tumor showed that adenocarcinoma was mainly present in the submucosa and muscularis propria, while the carcinoma-involved region of the mucosal layer had mucosal colonization representing the spread of metastatic tumor cells along the basement membrane of preexisting crypts and/or villi. The right ovarian tumor proved to be moderately differentiated adenocarcinoma that was positive for cytokeratin 20 and negative for cytokeratin 7 staining, indicating metastasis from the colorectal cancer. The rectal and ovarian tumors were similar to transverse colon cancer in architectural and cytological atypia. Both adenocarcinomas of the transverse colon and rectum were negative for p53 in immunohistochemical staining and *RAS* wild type in genetic assessment. These findings support a possible diagnosis of rectal and ovarian metastasis from the primary transverse colon cancer. The patient recovered well after surgery, and neither relapse nor metastasis was observed 18 months after surgery.

**Conclusion:**

Distinguishing primary from metastatic colorectal cancer can be challenging, but a comprehensive evaluation of histological features, clinical history, and tumor distribution can enable making a correct diagnosis and implementing optimal treatment.

## Background

Recent progress in multidisciplinary treatments has prolonged the survival of patients with metastatic colorectal cancer [1]. Consequently, the chances of encountering rare metastatic sites may be increased [2]. Colorectal metastases from primary colorectal cancers are rare, and little is known about their epidemiological aspects or the optimal diagnostic and therapeutic strategies. We herein report a case of suspected metachronous metastasis to the rectum from primary transverse colon cancer.

## Case presentation

A 65-year-old woman presented with abdominal pain and hypophagia for 2 weeks. She had a medical history of alcoholic pancreatitis. A computed tomography (CT) scan of her abdomen and pelvis revealed thickening of the transverse colon wall. Colonoscopy showed an ulcerated tumor in the transverse colon, and histological analysis indicated moderately differentiated adenocarcinoma. Serum carcinoembryonic antigen (CEA) levels were normal, and no sites of distant metastasis were reported on preoperative examination. She underwent a laparoscopic extended right hemicolectomy using the non-touch isolation technique. Histopathological examination of the specimen revealed a moderately differentiated adenocarcinoma invading the subserosal layer with low venous invasion (v1) and no lymphatic invasion (ly0). The surgical margins were negative. A total of 92 lymph nodes were removed, of which none showed metastases. The tumor was diagnosed as stage IIA (T3, N0, M0) according to the International Union Against Cancer tumor, node, and metastasis (TNM) classification (7th edition) [3]. The patient had an uneventful recovery and was on regular follow-up every 3 months without adjuvant chemotherapy.

**Fig. 1:**
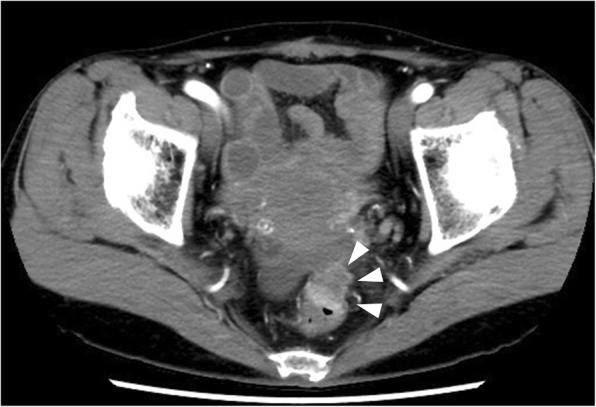
Computed tomography scan of the patient’s abdomen and pelvis showing a tumor 20 mm in size along the middle rectal wall and expanding into the pelvis (arrowhead)

**Fig. 2:**
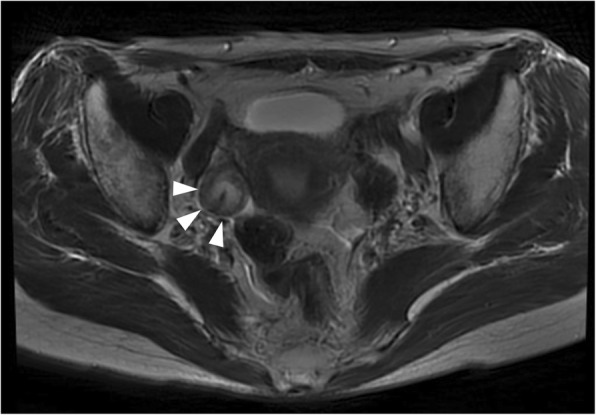
Magnetic resonance imaging revealed a right ovarian mass 25 mm in diameter with a solid component (arrowhead)

**Fig. 3:**
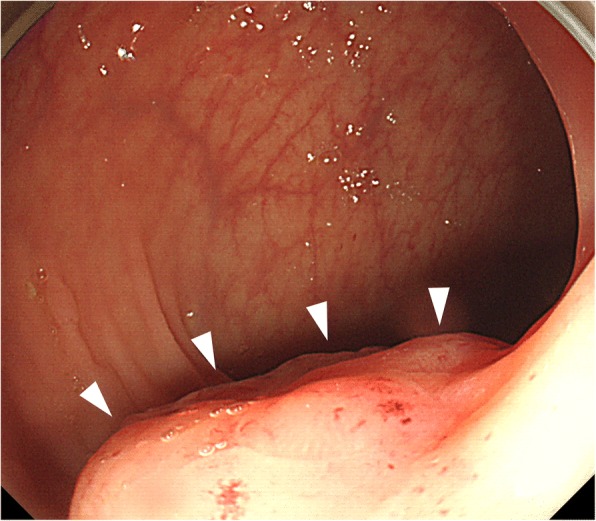
Colonoscopy showing a superficial elevated lesion in the middle rectum (arrowhead)

Fifteen months after her colectomy, a CT scan demonstrated a tumor in the antero-lateral rectal wall that was 20 mm in diameter, and a right ovarian tumor that was 25 mm in diameter (Fig. 1). The ovarian tumor was a cystic mass with a solid component on magnetic resonance imaging (Fig. 2). Colonoscopy revealed a superficial elevated lesion in the middle rectum that was shown by histological examination to be moderately differentiated adenocarcinoma (Fig. 3). Serum CEA levels were normal. These findings were indicative of rectal cancer with ovarian metastasis, or double primary cancer of the rectum and ovary. A diagnostic and therapeutic laparoscopy detected no disseminated peritoneal metastases or liver metastases. Intraoperative cytological examination of the peritoneal lavage was negative for carcinoma. An operative rapid pathological diagnosis of the resected right ovarian tumor indicated metastasis of the colorectal cancer. Thus, we performed laparoscopic low anterior resection preserving the left colic artery with partial resection of the vagina, and bilateral adnexectomy.

**Fig. 4:**
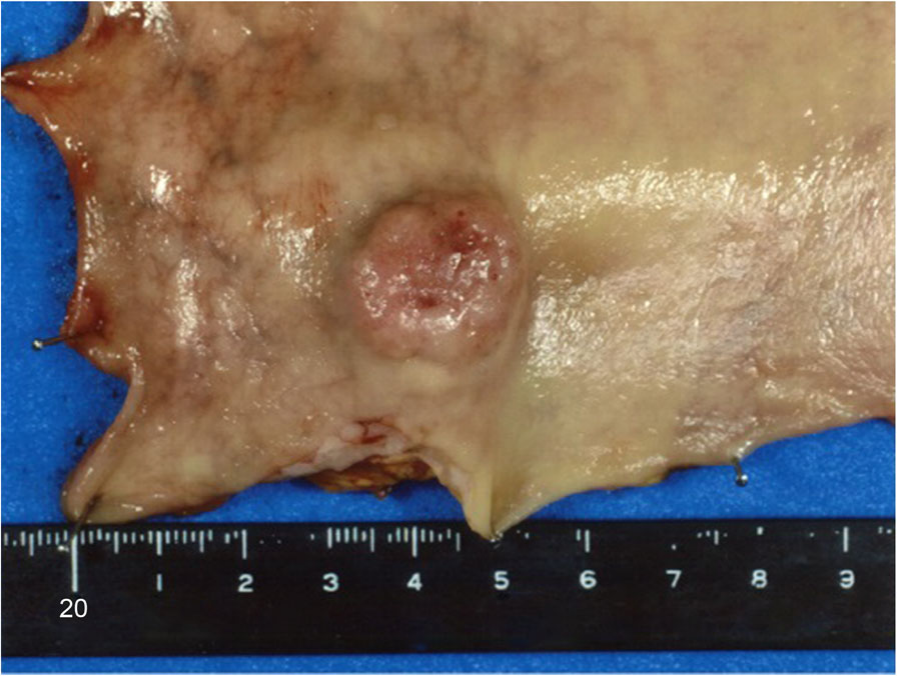
Macroscopic findings from resected rectal specimens. The resected rectal tumor measured 20 × 18 mm

**Fig. 5:**
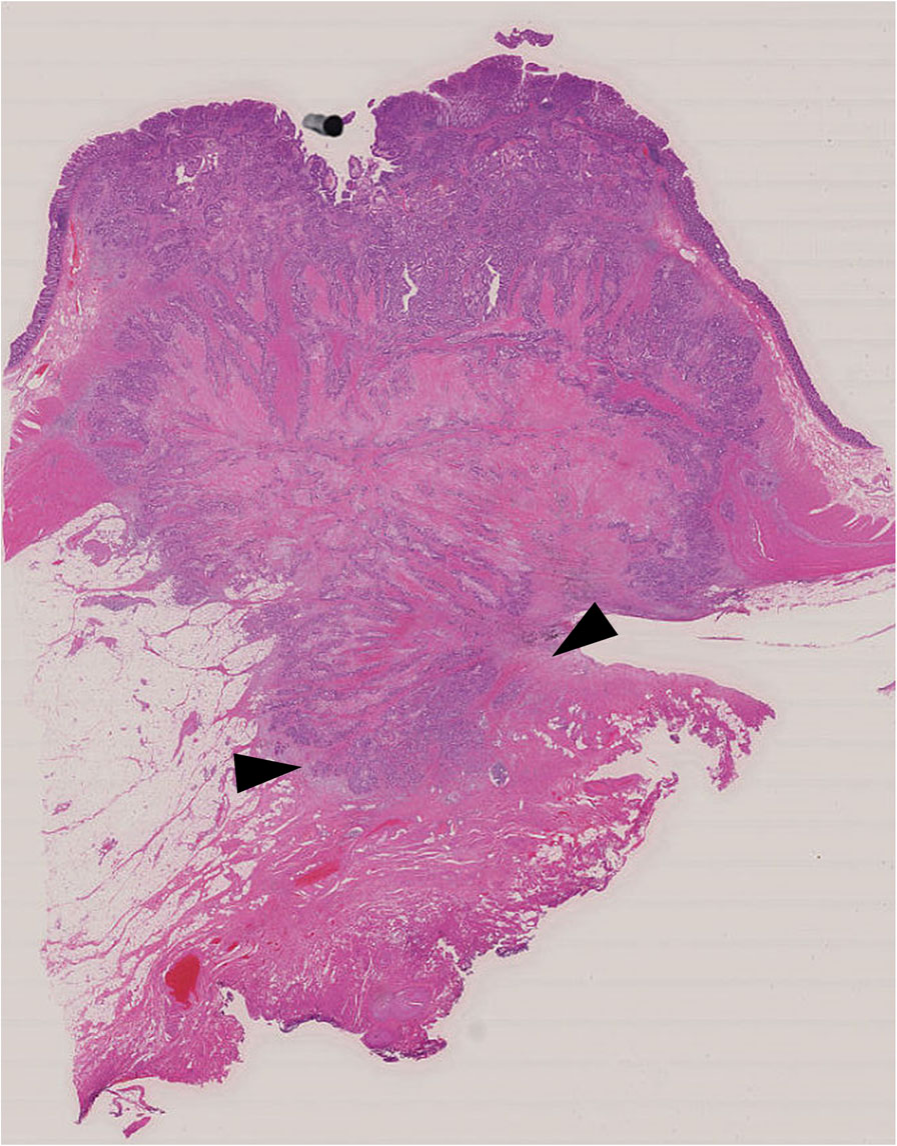
Histological examination of the resected specimens from the rectum (HE Loupe image). The viable adenocarcinoma was mainly present in the submucosa and muscularis propria with a small range of invasion to the vagina (arrowhead). HE, hematoxylin and eosin

**Fig. 6:**
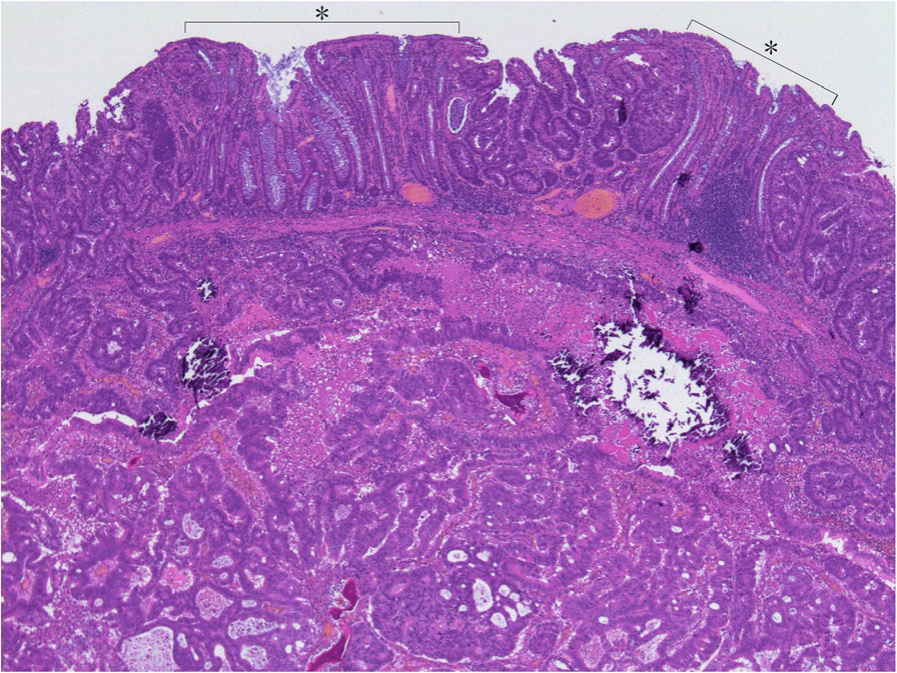
Histological examination of resected specimens from the rectum (HE staining; magnification × 40). The tumor-involved region of the mucosal layer showed a normal grand ductal structure resembling a floating island, which was indicative of mucosal colonization (*). HE, hematoxylin and eosin

**Fig. 7:**
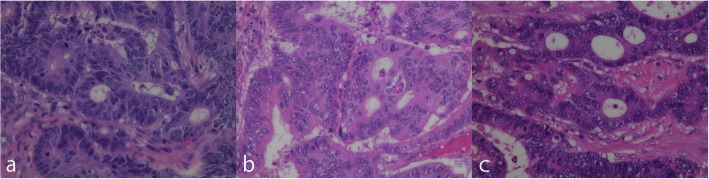
Histological comparison of resected specimens from the transverse colon (**a**), rectum (**b**), and right ovary (**c**) (HE staining; magnification × 200). Rectal and ovarian tumors shared many similarities with the transverse colon cancer in terms of architectural and cytological atypia. HE, hematoxylin and eosin

**Fig. 8:**
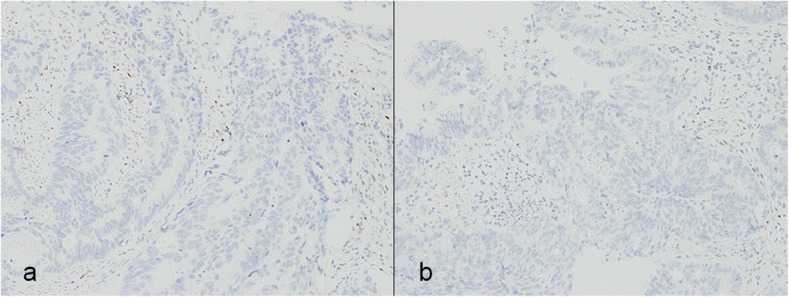
For immunohistochemical staining, both adenocarcinomas of the transverse colon (**a**) and rectum (**b**) were negative p53 immunohistochemical staining. This was a supportive finding that suggested both tumors were of the same origin

The resected rectal tumor measured 20 × 18 mm (Fig. 4). Histological examination demonstrated a moderately differentiated adenocarcinoma that had invaded the vagina and formed a metastasis in one of 32 lymph nodes. High venous invasion (v3) and low lymphatic invasion (ly1) were observed. The adenocarcinoma was mainly present in the submucosa and muscularis propria with a small range of invasion to the vagina (Fig. 5), while the carcinoma-involved region of the mucosal layer had mucosal colonization representing the spread of metastatic tumor cells along the basement membrane of preexisting crypts and/or villi (Fig. 6) [4]. There was no adenomatous precursor at the edge of the tumor, and surgical margins were negative. The right ovarian tumor was moderately differentiated adenocarcinoma that was positive for cytokeratin (CK) 20 and negative for CK7 immunohistochemical staining, indicating metastasis of colorectal cancer. The rectal and ovarian tumors shared high similarities with transverse colon cancer in architectural and cytological atypia (Fig. 7). Both adenocarcinomas of the transverse colon and rectum were negative for p53 immunohistochemical staining and *RAS* wild type in genetic assessment (Fig. 8). These findings support a diagnosis of rectal and ovarian metastases from primary transverse colon cancer.

The patient recovered well after surgery, and adjuvant chemotherapy was decided after a multidisciplinary meeting. She was treated with eight cycles of oxaliplatin and capecitabine, and neither relapse nor metastasis has been observed 18 months after surgery.

### Discussion

The incidence of colorectal metastasis from primary colorectal cancer is rare, and distinguishing primary from metastatic colorectal cancer can be challenging. To our knowledge, only three reports in English and six in Japanese have described suspected cases of colorectal metastasis of colorectal cancer [5–12]. We reviewed a total of eight patients, including seven out of the abovementioned nine patients with detailed clinical information as well as our own (Table 1). The patients were three males and five females, with a median age of 66.5 years (range 52–88 years). Ascending colon cancer was the most common primary tumor (*n* = 3), followed by tumors of the sigmoid (*n* = 2), transverse colon (*n* = 2), and cecum (*n* = 1). Metastatic colorectal lesions were located in the rectum in all cases. In terms of other metastatic sites, seven patients had other metastases (lung, liver, spleen, abdominal wall, axilla, and ovary) and one had no other metastases.

**Table 1:** Reported cases of colorectal metastasis from primary colorectal cancer

No.	First author (year) [reference in this manuscript]	Sex	Age (years)	Location of primary lesion	Histology of primary cancer	Duration^1^ (months)	Location of metastatic lesion	Other sites of metastasis	Prognosis (after diagnosis of the colorectal metastasis)
1	Kalaitzis (2010) [6]	F	72	Sigmoid colon	mod, mp, n (−)	12	Rectum	Abdominal wall	NA
2	Sasaki (2010) [7]	F	52	Sigmoid colon	mod, ss, ly1, v0, n (+)	12	Rectum	Liver/lung	Death (34 months)
3	Takashima (2010) [8]	F	80	Ascending colon	mod, se, ly1, v2, n (−)	19	Rectum	Lung	Alive (14 months)
4	Murakami (2011) [9]	F	88	Ascending colon	mod, se, ly1, v1, n (+)	3	Rectum	None	NA
5	Mishima (2013) [10]	M	65	Transverse colon	well, ss, ly1, v1, n (+)	27	Rectum	Liver	Alive (6 months)
6	Shimazaki (2014) [11]	M	68	Cecum	mod, ss, n (+)	23	Rectum	Liver, spleen	Alive (60 months)
7	Lucke-Wold (2017) [12]	M	56	Ascending colon	mod-muc, ss, n (+)	36	Rectum	Axilla	Death (about 2 years)
8	Our case	F	65	Transverse colon	mod, ss, ly0, v1, n (−)	15	Rectum	Ovary	Alive (18 months)

*F* female, *n* lymph node metastasis, *ly* lymphatic invasion, *NA* not available, *M* male, *se* extraserosal invasion, *mod* moderately differentiated adenocarcinoma, *mp* invasion into muscularis propia, *muc* mucinous adenocarcinoma, *ss* subserosal invasion, *v* venous invasion, *well* well-differentiated adenocarcinoma

^1^Duration before detection of metastatic tumor

A previous study of 278,208 malignancies in a nationwide Japanese pathologic autopsy database from 1990 to 2003 identified 18,252 case with metastatic colorectal cancer. Of these, 1302 (7.1%) were from primary colorectal cancer [13]. The database does not include information about the suspected metastatic pathway (hematogenous, lymphogenous, direct invasion, or dissemination), but most metastatic tumors in the database are attributed to direct invasion or disseminated metastasis, reflecting late-stage disease. However, the number of reported clinical cases might not represent the actual incidence of colorectal metastasis from colorectal cancer. One possible reason for the rarity of reported colorectal metastasis cases is that most cases occur as part of systemic advanced disease, for which surgical resection will not be performed [14]. The other reason is that diagnosing metastasis is difficult [15, 16]. Although gastrointestinal metastatic carcinoma usually represents a submucosal tumor, differential diagnosis (as primary disease or metastasis) becomes difficult if the tumor invades the mucosal layer. Estrella et al. argued that metastatic carcinomas involving the mucosal surface frequently mimic second primaries, so histologic features cannot reliably distinguish metastatic from primary carcinoma [4]. Additionally, primary colorectal cancers resembling submucosal tumors have been reported in previous studies [17, 18]. This explains why colorectal metastases may be misdiagnosed and treated as primaries.

Distinguishing primary from metastatic colorectal cancer can be challenging, but a comprehensive evaluation of histological features, clinical history, and tumor distribution enables making the correct diagnosis and implementing the optimal treatments [4]. In this case, we suspected a metachronous metastasis for the following reasons. First, histological findings demonstrated that the adenocarcinoma was mainly present in the submucosa and muscularis propria with mucosal colonization, which is a supportive finding for metastatic carcinoma, as this indicates a low possibility of primary rectal cancer and implantation of the transverse colon cancer. Second, the rectal and ovarian tumors were very similar to the transverse colon cancer in terms of architectural and cytological atypia, further suggesting metastasis of primary transverse colon cancer. Third, there was no adenomatous precursor at the tumor edge, indicating metastatic cancer. Fourth, both adenocarcinomas of the transverse colon and rectum were negative for p53 in immunohistochemical staining and *RAS* wild type in genetic assessment, indicating metastatic rectal cancer from primary transverse colon cancer.

In reviewed cases, the exact mechanism of colorectal metastasis from primary colorectal cancer has not been fully elucidated; thus, the possibility of hematogenous or lymphogenous spread remains. We suspected hematogenous or lymphogenous metastatic pathways to the rectum from the primary transverse colon cancer because the carcinoma was mainly located in submucosa and muscularis propria; however, there was no additional evidence than tumor localization. In this case, the rectal tumor penetrated into a small range of the subserosal layer with invasion to the vagina; additionally, there was a metastatic ovarian carcinoma. Peritoneal metastatic invasion primarily toward the deep layer of the rectal wall cannot be ruled out. The metastatic colorectal lesions were located in the rectum in all reviewed cases, and this suggests that peritoneal spread to Douglas’s pouch or the rectovesical pouch is a possible pathway for colorectal metastasis from primary colorectal cancer.

Considering that the majority of reported cases had multiple metastases, the existence of other metastatic lesions might be a risk factor for colorectal metastasis. Therefore, more attention should be paid to colorectal lesions when other metastatic sites have been identified. Colorectal metastases usually represent late-stage disease and have poor prognoses; however, prolonged survival after surgery and complementary therapy can be achieved in some patients [14]. In this case, neither relapse nor metastasis has been observed 18 months after surgery. The follow-up periods varied too widely to evaluate patient prognosis in reviewed cases. Therefore, additional studies are needed to better understand this rare metastasis and to determine the optimal therapeutic strategies.

## Conclusions

Distinguishing primary from metastatic colorectal cancer can be challenging, but a comprehensive evaluation of histological features, clinical history, and tumor distribution can enable making a correct diagnosis and implementing optimal treatment.

## Abbreviations

CEA: Carcinoembryonic antigen
CK: Cytokeratin
CT: Computed tomography
TNM: Tumor, node, and metastasis
